# Profile of the JACC Study

**DOI:** 10.2188/jea.15.S4

**Published:** 2005-05-16

**Authors:** Akiko Tamakoshi, Takesumi Yoshimura, Yutaka Inaba, Yoshinori Ito, Yoshiyuki Watanabe, Katsuhiro Fukuda, Hiroyasu Iso

**Affiliations:** 1 Department of Preventive Medicine/Biostatistics and Medical Decision Making, Nagoya University Graduate School of Medicine.; 2 Fukuoka Institute of Health and Environmental Sciences.; 3 Department of Epidemiology and Environmental Health, Juntendo University School of Medicine.; 4 Department of Public Health, Fujita Health University School of Health Sciences.; 5 Department of Epidemiology for Community Health and Medicine, Kyoto Prefectural University of Medicine Graduate School of Medical Science.; 6 Department of Public Health, Kurume University School of Medicine.; 7 Department of Public Health Medicine, Institute of Community Medicine, University of Tsukuba.

**Keywords:** JACC Study, profile

## Abstract

BACKGROUND: A large-scale population-based cohort study would offer the best evidence of a relation between lifestyle and cancer.

METHODS: The Japan Collaborative Cohort Study (JACC Study) for Evaluation of Cancer Risk sponsored by the Ministry of Education, Science, Sports and Culture of Japan (Monbusho) was established and carried out from 1988 to 1990 in 45 areas in Japan. Epidemiological information, such as demographic information, past medical history, exercise/sports activities engaged in, frequency of food intake, smoking and alcohol drinking status and so on, was collected by a self-administered questionnaire. Blood samples were collected from each participant at screening in 37 out of 45 areas to investigate risk factors of cancer relating to biochemical substances in blood. Collected sera were divided into 3-5 tubes (100-500µL per tube) and stored at -80°C. Additionally, about 5 years after the baseline survey, an interim survey regarding lifestyle changes was conducted in 31 areas. We followed the study subjects for mortality, move-outs, and cancer incidence, if possible (in 24 areas).

RESULTS: There were 127,477 participants (54,032 men and 73,445 women) registered in the study. Of these, 110,792 subjects (46,465 men and 64,327 women), aged 40 to 79 years at baseline, were eligible for follow-up. Sera were stocked from 39,242 subjects, and interim survey was carried out on 46,650 subjects.

CONCLUSIONS: The JACC Study provides useful evidence for cancer prevention in Japan.

To confirm the association of lifestyle factors with cancer, large-scale population-based studies are believed to give the best evidence. In Japan before about 1985, however, there was only one large-scale cohort study running to evaluate cancer risk in regard to lifestyle, which was called the Six-Prefecture Cohort Study.^1^ Though the study demonstrated some useful information for preventing cancer, because of rapid lifestyle change, we conducted another large-scale population-based study in the late 1980s^2^^,^^3^ to reveal risk factors and to provide cancer prevention strategies. In this study, not only traditional epidemiological data such as socio-demographic or lifestyle factors but also sera (from one-third of whole subjects) were collected and stocked to investigate risk factors of cancer relating to biochemical substances in blood. Moreover, to consider the magnitude of lifestyle changes in relation to cancer risk, an interim survey was conducted on about 40% of the study subjects. This paper reports the profile of this cohort study, the Japan Collaborative Cohort Study (JACC Study) for Evaluation of Cancer Risk sponsored by the Ministry of Education, Science, Sports and Culture of Japan (Monbusho).

## METHODS

### Study Participants

**Figure 1.**
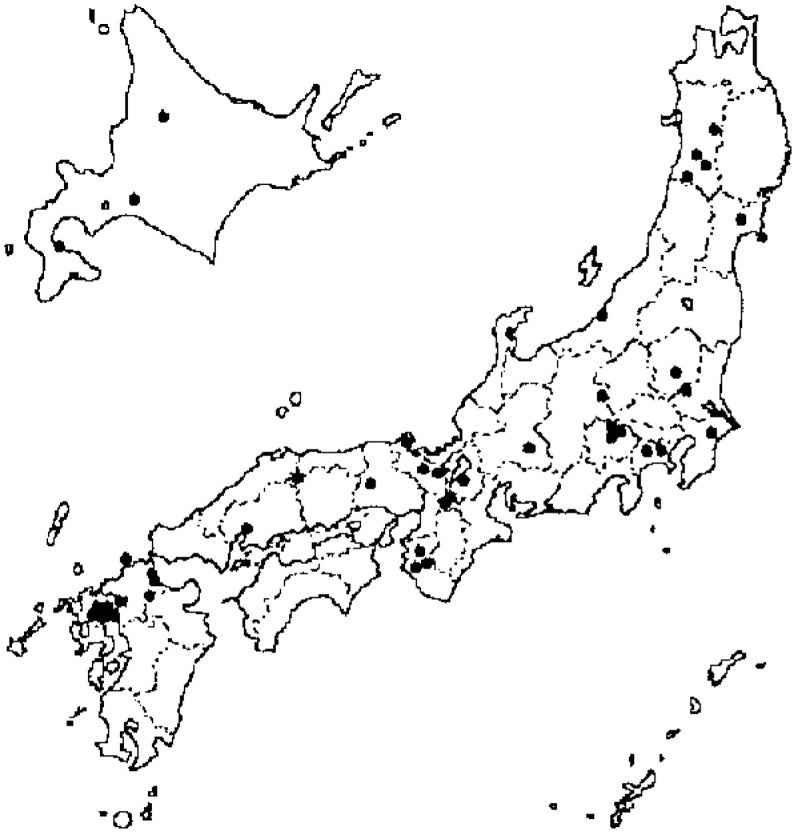
Geographical distribution of study areas.

From 1988 through 1990, we established the JACC study in 45 areas in Japan: 3 towns in the Hokkaido district, 5 towns in the Tohoku district, 5 towns in the Kanto district, 1 city, 3 towns and 2 villages in the Chubu district, 8 towns and 2 villages in the Kinki district, 1 city and 1 town in the Chugoku district, and 4 cities, 9 towns and 1 village in the Kyushu district (none in the Shikoku district) (Figure 1). This was a multicenter-collaborative study in which 24 institutions voluntarily participated. The recruitment of the study subjects fell to each investigator, who had the responsibility to construct a cohort in each area. In 22 out of 45 areas, all residents living in a given target area (not always equal to the whole area, but somewhat small and describable district(s) in it) were regarded as study subjects, and questionnaires were supplied. In 20 areas, those who had undertaken a basic health examination that was conducted under the Health and Medical Service Law for the Aged were invited to participate in the study. In 2 areas, the study subjects consisted of health examination examinees plus volunteers. In 1 area, subjects were defined based on the health checkup for atomic bomb survivors.

### Data Collection and Informed Consent Questionnaire

To collect epidemiological information, a self-administered questionnaire was used. Questionnaire items included: such information as demographic information, past medical history, family medical history, health condition one year prior to entry, exercise/sports activities engaged in, frequency of food intake and preference for salty and fatty foods, smoking and alcohol drinking status, health check-up history, occupation, residential area, education, behavioral attitude/stress, and reproductive history for women. Individual informed consent to participate in the study was obtained in 36 out of 45 areas (written consent in 35 areas and oral consent in 1 area). In 9 areas, however, group consent from the head of the area was obtained. All information was entered into a computer using the same format, under the responsibility of each investigator, and then sent to the central secretariat of the JACC study (Department of Preventive Medicine/Biostatistics and Medical Decision Making, Nagoya University Graduate School of Medicine) without any subject being identified.

### Serum sampling

Blood samples from each participant were also taken at screening in 37 out of 45 areas. Collected sera were divided into 3-5 tubes (100-500µL per tube) and stored at -80°C, primarily at the JACC Study central office. Written consent was obtained in 25 areas, and oral consent was obtained in six. In 1 area, posters were used to inform the public and allow people to opt out, while in another 5 areas consent was obtained only from the head of the area.

### Interim survey

About 5 years after the baseline survey, we conducted an interim survey on lifestyle factors in 31 areas, though not all the participants in each area were included in the result. The research was done using a self-administered questionnaire, included items on demographic information, past medical history, family cancer history in these 5 years, exercise/sports activities, frequency of food intake and change of intake compared with 5 years before, hair dye use, smoking and alcohol drinking status, and health checkup history. Individual informed consent was obtained in 25 areas (written consent in 24 areas and oral consent in 1 area), and group consent from the head of the area was obtained in 6 areas. No blood samples were collected this time.

### Data Cleaning

To maintain uniformity in the responses, we checked and revised the answers as follows: (1) when answered values were out of the range, they were regarded as a missing value; (2) when answers were contradictory among several related questions, they were regarded as missing values or corrected to consistent responses if possible.

### Follow-up

The date and cause of death were annually or biannually confirmed, with the permission of the director-general of the Prime Minister’s Office (Ministry of Public Management, Home Affairs, Post and Telecommunications). The date of move-out from the study area was also annually verified by the investigator in each area by reviewing population-register sheets of the cohort members. In 24 out of 45 areas, we also collected data on cancer incidence, incident date and site, through the local cancer registry or by reviewing local major hospital records.

### Ethical Review

Our entire study design, which comprised singular and collective use of epidemiologic data and biological materials (serum only), was approved in 2000 by the Ethical Board at Nagoya University School of Medicine, where the central secretariat of the JACC study is located.

## RESULTS

There were 127,477 participants (54,032 men and 73,445 women) registered in the study. Of these, we followed 110,792 subjects (46,465 men and 64,327 women), aged 40 to 79 years at baseline. The average response rate among 17 areas which included all living residents as the subjects was 83% (In 3 of 22 areas, the number of residents was unknown, and in 2 areas the target area proved to be smaller than the reported overall area, so they were excluded from the calculation). Table 1 shows the age and sex distribution of the study subjects. Women/men ratio was constantly greater than 1.0 (1.38 for all subjects), and the highest ratio was observed in the 65-69 (1.56) age group.

**Table 1.** Age distribution of cohort members at baseline by district.

	Age at entry (year)								Total

	40-44	45-49	50-54	55-59	60-64	65-69	70-74	75-79	

	Men								

Total	6002	5806	6322	7695	8429	5518	4024	2669	46465

Hokkaido	191	182	212	267	284	200	86	43	1465
Tohoku	810	627	802	1052	1269	892	492	294	6238
Kanto	624	630	610	660	753	572	323	219	4391
Chubu	2444	2252	2172	2423	2504	1708	1299	918	15720
Kinki	958	908	1148	1454	1422	999	653	458	8000
Chugoku	225	379	456	891	1256	593	774	520	5094
Kyushu	750	828	922	948	941	554	397	217	5557

	Women								

Total	7557	7926	9108	10816	11114	8602	5557	3647	64327

Hokkaido	310	310	433	436	382	257	93	37	2258
Tohoku	961	963	1416	1676	1670	1135	602	372	8795
Kanto	814	765	818	878	907	821	430	271	5704
Chubu	2497	2351	2464	2667	2945	2367	1687	1158	18136
Kinki	1255	1221	1510	1791	1569	1301	877	626	10150
Chugoku	306	799	835	1484	2201	1810	1299	860	9594
Kyushu	1414	1517	1632	1884	1440	911	569	323	9690

We stocked sera of 39,242 subjects (42,249 when not restricted to those aged 40 to 79 at baseline). The peak donation rate was in the 60-64 age group among men and in the 55-59 age group among women (Table 2).

**Table 2.** Age distribution at baseline of cohort members who donated blood sample.

	Age at entry (year)																Total	
	
	40-44		45-49		50-54		55-59		60-64		65-69		70-74		75-79			
	Men																	
	1580	(26.3)	1424	(24.5)	1770	(28.0)	2486	(32.3)	2979	(35.3)	1950	(35.3)	1090	(27.1)	560	(21.0)	13839	(29.8)
																		
Hokkaido	124		122		123		179		188		122		52		14		924	
Tohoku	391		286		391		517		664		458		223		124		3054	
Kanto	253		222		251		302		424		369		176		108		2105	
Chubu	384		336		376		513		670		426		235		78		3018	
Kinki	232		257		350		528		519		351		279		162		2678	
Chugoku	20		37		50		106		158		66		80		57		574	
Kyushu	176		164		229		341		356		158		45		17		1486	

	Women																	
Total	3037	(40.2)	3157	(39.8)	4086	(44.9)	4996	(46.2)	4657	(41.9)	3147	(36.6)	1567	(28.2)	756	(20.7)	25403	(39.5)
																		
Hokkaido	210		206		280		296		255		165		53		14		1479	
Tohoku	454		458		811		994		954		590		276		163		4700	
Kanto	436		381		469		525		569		522		251		139		3292	
Chubu	615		618		754		874		1035		684		292		89		4961	
Kinki	520		579		722		910		710		559		369		196		4565	
Chugoku	46		135		174		267		393		277		248		137		1677	
Kyushu	756		780		876		1130		741		350		78		18		4729	

Parcentages of those donating blood samples in parentheses.

The interim survey was done on 51,723 subjects, of whom 46,650 (18,312 men and 28,338 women) were aged 40 to 79 years at the baseline survey (Table 3). The highest participation rate was observed among the 60-64 age group at baseline.

**Table 3.** Age distribution of cohort members included in interim research.

	Age at entry (year)																Total	
	
	40-44		45-49		50-54		55-59		60-64		65-69		70-74		75-79			
	Men																	
Total	2115	(35.2)	2126	(36.6)	2477	(39.2)	3339	(43.4)	3788	(44.9)	2218	(40.2)	1502	(37.3)	747	(28.0)	18312	(39.4)
Hokkaido	77	(40.3)	87	(47.8)	104	(49.1)	129	(48.3)	149	(52.5)	81	(40.5)	32	(37.2)	14	(32.6)	673	(45.9)
Tohoku	349	(43.1)	292	(46.6)	370	(46.1)	477	(45.3)	574	(45.2)	365	(40.9)	158	(32.1)	72	(24.5)	2657	(42.6)
Kanto	243	(38.9)	264	(41.9)	232	(38.0)	257	(38.9)	264	(35.1)	175	(30.6)	109	(33.7)	69	(31.5)	1613	(36.7)
Chubu	543	(22.2)	408	(18.1)	438	(20.2)	616	(25.4)	622	(24.8)	388	(22.7)	158	(12.2)	24	(2.6)	3197	(20.3)
Kinki	208	(21.7)	201	(22.1)	357	(31.1)	493	(33.9)	513	(36.1)	347	(34.7)	239	(36.6)	125	(27.3)	2483	(31.0)
Chugoku	185	(82.2)	282	(74.4)	362	(79.4)	715	(80.2)	1011	(80.5)	480	(80.9)	552	(71.3)	330	(63.5)	3917	(76.9)
Kyushu	510	(68.0)	592	(71.5)	614	(66.6)	652	(68.8)	655	(69.6)	382	(69.0)	254	(64.0)	113	(52.1)	3772	(67.9)

	Women																	
Total	3031	(40.1)	3450	(43.5)	4085	(44.9)	5044	(46.6)	5503	(49.5)	3867	(45.0)	2183	(39.3)	1175	(32.2)	28338	(44.1)

Hokkaido	144	(46.5)	157	(50.6)	213	(49.2)	216	(49.5)	206	(53.9)	110	(42.8)	39	(41.9)	8	(21.6)	1093	(48.4)
Tohoku	412	(42.9)	423	(43.9)	662	(46.8)	718	(42.8)	749	(44.9)	460	(40.5)	182	(30.2)	104	(28.0)	3710	(42.2)
Kanto	315	(38.7)	319	(41.7)	346	(42.3)	364	(41.5)	326	(35.9)	282	(34.3)	161	(37.4)	97	(35.8)	2210	(38.7)
Chubu	721	(28.9)	673	(28.6)	723	(29.3)	805	(30.2)	856	(29.1)	527	(22.3)	141	(8.4)	28	(2.4)	4474	(24.7)
Kinki	280	(22.3)	292	(23.9)	438	(29.0)	546	(30.5)	607	(38.7)	470	(36.1)	301	(34.3)	209	(33.4)	3143	(31.0)
Chugoku	262	(85.6)	656	(82.1)	695	(83.2)	1237	(83.4)	1834	(83.3)	1452	(80.2)	969	(74.6)	551	(64.1)	7656	(79.8)
Kyushu	897	(63.4)	930	(61.3)	1008	(61.8)	1158	(61.5)	925	(64.2)	566	(62.1)	390	(68.5)	178	(55.1)	6052	(62.5)

Parcentages of those participating the interim research in parentheses.

## DISCUSSION

In this paper, we reported the profile of the JACC Study, started in 1988-90. This study is unique in the following points:

(1) A large-scale prospective study involving 110,792 healthy subjects aged 40-79 years old from across Japan, it considered risk factors and prevention strategies of cancer from a national view point.

(2) The study stored sera from 1/3 of the subjects, making it possible to investigate cancer risk factors of related to biochemical substances in blood.

(3) Cancer incidence data were obtained from 24 out of 45 areas, which allowed us to find out not only the promoting factors but also the initiating factors of cancer.

(4) The interim survey on 1/3 of the subjects made it possible to consider the effect of changing risk factors in relation to cancer.

Follow-up was carried out until 1999, and would be extended until 2003. From the JACC Study, useful evidence for cancer prevention in Japan will surely be obtained.

## MEMBER LIST OF THE JACC STUDY GROUP

The present investigators involved, with the co-authorship of this paper, in the JACC Study and their affiliations are as follows: Dr. Akiko Tamakoshi (present chairman of the study group), Nagoya University Graduate School of Medicine; Dr. Mitsuru Mori, Sapporo Medical University School of Medicine; Dr. Yutaka Motohashi, Akita University School of Medicine; Dr. Ichiro Tsuji, Tohoku University Graduate School of Medicine; Dr. Yosikazu Nakamura, Jichi Medical School; Dr. Hiroyasu Iso, Institute of Community Medicine, University of Tsukuba; Dr. Haruo Mikami, Chiba Cancer Center; Dr. Yutaka Inaba, Juntendo University School of Medicine; Dr. Yoshiharu Hoshiyama, University of Human Arts and Sciences; Dr. Hiroshi Suzuki, Niigata University School of Medicine; Dr. Hiroyuki Shimizu, Gifu University School of Medicine; Dr. Hideaki Toyoshima, Nagoya University Graduate School of Medicine; Dr. Kenji Wakai, Aichi Cancer Center Research Institute; Dr. Shinkan Tokudome, Nagoya City University Graduate School of Medical Sciences; Dr. Yoshinori Ito, Fujita Health University School of Health Sciences; Dr. Shuji Hashimoto, Fujita Health University School of Medicine; Dr. Shogo Kikuchi, Aichi Medical University School of Medicine; Dr. Akio Koizumi, Graduate School of Medicine and Faculty of Medicine, Kyoto University; Dr. Takashi Kawamura, Kyoto University Center for Student Health; Dr. Yoshiyuki Watanabe, Kyoto Prefectural University of Medicine Graduate School of Medical Science; Dr. Tsuneharu Miki, Graduate School of Medical Science, Kyoto Prefectural University of Medicine; Dr. Chigusa Date, Faculty of Human Environmental Sciences, Mukogawa Women’s University ; Dr. Kiyomi Sakata, Wakayama Medical University; Dr. Takayuki Nose, Tottori University Faculty of Medicine; Dr. Norihiko Hayakawa, Research Institute for Radiation Biology and Medicine, Hiroshima University; Dr. Takesumi Yoshimura, Fukuoka Institute of Health and Environmental Sciences; Dr. Akira Shibata, Kurume University School of Medicine; Dr. Naoyuki Okamoto, Kanagawa Cancer Center; Dr. Hideo Shio, Moriyama Municipal Hospital; Dr. Yoshiyuki Ohno, Asahi Rosai Hospital; Dr. Tomoyuki Kitagawa, Cancer Institute of the Japanese Foundation for Cancer Research; Dr. Toshio Kuroki, Gifu University; and Dr. Kazuo Tajima, Aichi Cancer Center Research Institute.

## References

[1] Hirayama T. Life-style and mortality: A large-scale census-based cohort study in Japan. Karger, Basel, 1990.

[2] Aoki K. Report by the Research Committee of the Ministry of Education, Science, Sports and Culture on evaluation of risk factors for cancer. J Epidemiol 1996;6:S107-S113. 8800281 10.2188/jea.6.3sup_107

[3] Ohno Y, Tamakoshi A; the JACC Study Group. Japan collaborative cohort study for evaluation of cancer risk sponsored by Monbusho (JACC study). J Epidemiol 2001; 11: 144-50. 11512570 10.2188/jea.11.144 PMC11735075

